# Radiologic and Clinical Bronchiectasis Associated with Autosomal Dominant Polycystic Kidney Disease

**DOI:** 10.1371/journal.pone.0093674

**Published:** 2014-04-18

**Authors:** Teng Moua, Ladan Zand, Robert P. Hartman, Thomas E. Hartman, Dingxin Qin, Tobias Peikert, Qi Qian

**Affiliations:** 1 Division of Pulmonary/Critical Care, Mayo Clinic Rochester, Rochester, Minnesota, United States of America; 2 Division of Nephrology, Mayo Clinic Rochester, Rochester, Minnesota, United States of America; 3 Department of Radiology, Mayo Clinic Rochester, Rochester, Minnesota, United States of America; University of Sao Paulo Medical School, Brazil

## Abstract

**Background:**

Polycystin 1 and 2, the protein abnormalities associated with autosomal dominant polycystic kidney disease (ADPKD), are also found in airway cilia and smooth muscle cells. There is evidence of increased radiologic bronchiectasis associated with ADPKD, though the clinical and functional implications of this association are unknown. We hypothesized an increased prevalence of both radiologic and clinical bronchiectasis is associated with APDKD as compared to non-ADPKD chronic kidney disease (CKD) controls.

**Materials and Methods:**

A retrospective case-control study was performed at our institution involving consecutive ADPKD and non-ADPKD chronic kidney disease (CKD) patients seen over a 13 year period with both chest CT and PFT. CTs were independently reviewed by two blinded thoracic radiologists. Manually collected clinical data included symptoms, smoker status, transplant history, and PFT findings.

**Results:**

Ninety-two ADPKD and 95 non-ADPKD CKD control patients were compared. Increased prevalence of radiologic bronchiectasis, predominantly mild lower lobe disease, was found in ADPKD patients compared to CKD control (19 vs. 9%, P = 0.032, OR 2.49 (CI 1.1–5.8)). After adjustment for covariates, ADPKD was associated with increased risk of radiologic bronchiectasis (OR 2.78 (CI 1.16–7.12)). Symptomatic bronchiectasis occurred in approximately a third of ADPKD patients with radiologic disease. Smoking was associated with increased radiologic bronchiectasis in ADPKD patients (OR 3.59, CI 1.23–12.1).

**Conclusions:**

Radiological bronchiectasis is increased in patients with ADPKD particularly those with smoking history as compared to non-ADPKD CKD controls. A third of such patients have symptomatic disease. Bronchiectasis should be considered in the differential in ADPKD patients with respiratory symptoms and smoking history.

## Introduction

Autosomal dominant polycystic kidney disease (ADPKD) characteristically manifests with progressive fluid filled renal cysts leading to end-stage renal disease in approximately 50% of patients [1], [2]. The contributing genetic defects are found in polycystin-1 and 2, two transmembrane regulatory proteins responsible for mechanoreception, cell polarization, and orientation [3], [4].

Multiple non-pulmonary extra-renal manifestations of ADPKD have been described [5], [6], [7]. Interestingly, polycystins are expressed in the cilia of both human airway epithelial [8] and airway smooth muscle cells [9]. Consequently, functional abnormalities in polycystins may result in radiological bronchiectasis due to decreased mucociliary clearance or impaired airway injury repair [8]. The functional and clinical significance of this radiologic association remains mostly unexplored.

In the current study we investigated the possible association of radiologic bronchiectasis with abnormal PFT and increased clinical pulmonary disease in a retrospective review of consecutive ADPKD patients seen at our institution as case patients compared to non-ADPKD CKD controls.

## Materials and Methods

IRB approval was obtained (Mayo Clinic IRB#: 09-002623) regarding study of patients giving written consent to have their stored medical records reviewed for the purposes of research. Clinical records of consecutive adult ADPKD patients seen at Mayo Clinic, Rochester, who underwent both high resolution chest computed tomography (HRCT) and pulmonary function testing (PFT) between 1998 and 2011, were reviewed. Patients were excluded if they had a known secondary etiology for clinical or radiologic bronchiectasis as reviewed in the available record, specifically cystic fibrosis (CF), prior mechanical airway obstruction, chest trauma or surgery resulting in focal lung injury, recurrent or severe pneumonia, or clinical immunodeficiencies such as common variable immunodeficiency (CVID) resulting in proclivity towards recurrent infection. Patients who underwent transplantation of any organ other than lung (solid and hematological) were included in the study. Non-ADPKD chronic kidney disease (CKD) patients who also underwent chest CT and PFT during the same study period were selected as consecutive unmatched controls. The most recent chest CT in the record was used if multiple studies were completed.

Collected demographics were manually obtained from the primary medical record for both study groups including age, gender, smoker status (active, former, and non-smoker), pack years, and transplant history. Laboratory data included stable GFR and creatinine within one year of the selected study CT. Clinical pulmonary diagnoses or symptoms of individual study patients at the time of CT were categorized in the following manner: Group 0 = None or no longstanding clinical pulmonary disease, Group 1 = idiopathic clinical bronchiectasis defined as presenting symptoms of chronic productive cough, dyspnea, and/or other constitutional symptoms such as fever, weight loss, not explained by a secondary or underlying pulmonary diagnosis, Group 2 = All other airways disease (COPD, asthma, bronchial or small airways disease), and Group 3 = All other parenchymal, pleural, or pulmonary vascular disease (acute infection, interstitial lung disease or fibrosis, granulomatous disease, malignant and non-malignant nodules or masses, pulmonary emboli, vasculitis, and pleural disease). For patients with multiple pulmonary diagnoses, idiopathic clinical bronchiectasis (Group 1) was selected first as a primary categorization if present followed by all other airways disease (Group 2), then all other parenchymal/pleural/pulmonary vascular disease (Group 3). Our rationale was to categorize or capture existing clinical bronchiectasis or airways disease in the setting of radiologic findings.

CT criteria for radiologic bronchiectasis included one or more of the following: 1) an enlarged bronchial diameter greater than that of the accompanying blood vessel (Signet ring sign), 2) failure of airway tapering at least 2 cm beyond the last branch point, or 3) visible airway within one centimeter of the lung periphery [10], [11], [12]. All selected CT scans were independently reviewed by two experienced thoracic radiologists (RH & TH) who were blinded to the presence of ADPKD and CKD with agreement on presence and severity of radiologic bronchiectasis by consensus.

Pulmonary function testing done within one year of the selected chest CT was categorized based on standard criteria [13], [14], [15] into one of four diagnostic findings: 1) Normal, 2) Obstructive, 3) Restrictive, or 4) Other (mixed restrictive and obstructive, non-specific pattern, and isolated low diffusing capacity for carbon monoxide (DLCO)); interpreted previously in the record by an experienced non-study pulmonologist. No further revision or reinterpretation of PFT findings was done by study investigators. If PFT was not available within one year of the selected scan, the most recent PFT in the clinical record was reviewed. Selected PFT measurements, including pre-bronchodilator percent predicted forced expiratory volume in 1 second (FEV_1_) and forced vital capacity (FVC), total lung capacity (TLC), forced expiratory flow at 25–75% of the FVC (FEF _25–75_), FEV1/FVC ratio, and diffusing capacity for carbon monoxide (DLCO) were also collected and compared between the two cohorts.

Statistical analysis was performed using JMP Software Version 9.4 (Cary, NC) with Chi-square or Fisher's exact test applied to proportional or categorical data, and a Two Sample T-test with 2-sided P value used for comparison of continuous or mean data. Chi-square and ANOVA were used to compare proportion and means among multiple groups. For predictors of a dichotomous outcome, univariable and multivariable logistic regression was applied adjusting for a priori selected covariates of age, gender, GFR, smoker status, and transplant history. Two-tailed P values<0.05 were considered statistically significant.

No external funding was involved in the hypothesis, study design, data collection, or analysis, of this work.

## Results

Ninety-two consecutive ADPKD patients and 95 consecutive non-ADPKD CKD patients underwent both chest CT and PFT and were ultimately included in the study and control groups, respectively. Comparison baseline demographic and clinical characteristics are presented in Table 1. Of the initial screening cohort fitting radiologic criteria for bronchiectasis, two were excluded from the ADPKD group secondary to history of prior severe pneumonia as likely causes of radiologic findings, and one was excluded from the CKD group due to concomitant cystic fibrosis diagnosis.

**Table 1:** Demographics and baseline characteristics.

	ADPKD (N = 92)	Control (N = 95)	P value
Characteristic			
Age, mean (SD)	59.84 (12.6)	61.59 (12.9)	0.35
Gender, M/F (%)	42/50 (46/54)	64/31 (67/33)	**0.003**
BMI, mean (SD)	29.21 (6.9)	30.06 (6.1)	0.37
Smoker Status;			0.35
Non-smoker N (%)	46 (50)	41 (43)	
Former N (%)	38(41)	52 (55)	
Active N (%)	8(9)	2(2)	
Pack years, mean (SD)	31.41 (20.7)	37.32 (29.5)	0.26
Creatinine, mean (SD)	2.10 (1.9)	1.93 (1.2)	0.47
GFR, mean (SD)	50.1 (28.7)	46.92 (25.4)	0.43
Radiologic bronchiectasis, N (%)	19 (21)	9 (9.1)	**0.032**
Clinical bronchiectasis, N (%)	6 (7)	3 (3)	0.32
Summary PFT dx			0.121
Normal, N (%)	42 (46)	29 (31)	
Obstructive, N (%)	26 (28)	30 (31)	
Restrictive, N (%)	6 (6)	13 (14)	
Other, N (%)	18 (20)	23 (24)	
FEV1 % predicted, mean (SD), range	79.28 (21.5), (36–119)	71.96 (24), (22–121)	**0.032**
FVC % predicted, mean (SD), range	86.78 (18.7), (36–134)	77.15 (22.6), (24–119)	**0.003**
FEV1/FVC, mean (SD), range	72.2 (10.1), (35.2–88.5)	72.3 (10.2), (33.9–95.2)	0.98
FEF 25–75 % predicted, mean (SD), range	67.2 (33.1), (13–152)	61 (36.8), (6–174)	0.23
TLC % predicted, mean (SD), range	96.25 (22.4), (53–164) (N = 52)	89.64 (20.1), (47–130) (N = 56)	0.112
DLCO % predicted, mean (SD), range	76.11 (19.3), (34–134) (N = 85)	68.24 (23.4), (17–125) (N = 80)	**0.022**
Transplant of any kind, N (%)	37 (40)	28 (29)	0.123
Time from Transplant to Study CT, months, median (range)	35.4 (−121–291) (N = 37)	31.4 (−30–163.5) (N = 28)	0.63

Compared to ADPKD there were more men in the consecutively selected CKD control group (67% vs. 46%, P = 0.003). Mean FEV_1_, FVC, and DLCO were statistically lower in the CKD group compared to ADPKD, though smoking status and summary PFT findings were not statistically different (P = 0.35 and P = 0.121, respectively). Frequency of organ transplantation of both solid and hematological origin was similar (P = 0.123). The majority of CT scans in both groups was obtained for assessment of clinical respiratory symptoms or previously established pulmonary disease, with none obtained for incidental findings found initially on lower lung cuts of abdominal CTs. In those whom underwent organ transplantation, 25% of reviewed CTs occurred prior to transplantation. The median time from date of transplant to CT was 35.4 (range −121 to 291) months, with no statistical difference between the two groups (P = 0.62).

Subgroup comparison data for baseline clinical features among APDKD patients with and without radiologic bronchiectasis is presented in Table 2. Smoking history was more prevalent in ADPKD patients with radiological bronchiectasis (74% vs. 44%, P = 0.04) without difference in other baseline characteristics.

**Table 2:** Subgroup Analysis of ADPKD with and without radiologic bronchiectasis.

	ADPKD Bronchiectasis (N = 19)	ADPKD No Bronchiectasis (N = 73)	P value
**Characteristic**			
Age, mean (SD)	62.63 (12.2)	59.1 (12.7)	0.28
Gender, M/F (%)	11/8 (58/42)	31/42 (42/58)	0.23
BMI, mean (SD)	27.52 (5.4)	29.66 (7.3)	0.24
Smoker status;			**0.021**
Non-smoker N (%),	5 (26)	41 (56)	
Former N (%)	12 (63)	26 (36)	
Active N (%)	2 (11)	6 (8)	
Creatinine, mean (SD)	1.62 (1.1)	2.23 (2.1)	0.24
GFR, mean (SD)	52.26 (22.7)	49.53 (30.2)	0.71
PFT dx			0.52
Normal, N (%)	8 (42)	34 (47)	
Obstructive, N (%)	6 (32)	20 (27)	
Restrictive, N (%)	0 (0)	6 (8)	
Other, N (%)	5 (26)	13 (18)	
FEV1 % predicted, mean (SD), range	81.42 (19.9), (39–108)	78.73 (22.1), (36–119)	0.63
FVC % predicted, mean (SD), range	87.68 (14.6), (53–105)	86.55 (19.7), (36–134)	0.81
FEV1/FVC (SD), range	72.32 (9.9), (55.6–86.5)	72.22 (10.3), (35.2–88.5)	0.96
FEF 25–75 % predicted (SD), range	60.5 (33.2), (15–136)	68.9 (33.1), (13–152)	0.33
TLC % predicted, mean (SD), range	99.7 (13.6), (79–122) (N = 10)	95.43 (24.1), (53–164) (N = 42)	0.59
DLCO % predicted, mean (SD), range	71.83 (19), (45–107) (N = 18)	77.25 (19.4), (34–134) (N = 67)	0.29
			
Transplant of any kind (N, %)	10 (53)	27 (37)	0.22
Time from Transplant to study CT, months, median (range)	67. 4 (−37–268)	22.8 (−120–292)	0.27

Radiologic changes of bronchiectasis were more frequent in ADPKD patients (19 (21%) vs. 9 (9%); P = 0.032, OR 2.49 (CI 1.1–5.8)), with no difference in prevalence of clinically symptomatic disease (6 (7%) vs. 3 (3%), P = 0.32) (Table 1.) Univariate logistic regression for selected covariates is presented in Table 3 for the whole cohort and Table 4 for subgroup analysis of the ADPKD group. The presence of ADPKD was associated with radiologic bronchiectasis, even after adjusting for age, gender, GFR, transplant history, and smoking by multivariate regression (OR 2.78, CI 1.16–7.12). Smoking history among ADPKD patients after adjustment for age, gender, GFR, and transplant history, was associated with increased risk of radiologic bronchiectasis (OR 4.79, CI 1.43–19.58) despite similar rates of smoking between ADPKD and CKD patients. Smoking was not associated with increased clinical disease among the two groups (ADPKD 5/6 (83%) vs. 41/86 (47%), P = 0.09 vs. Control 1/3 (33%) vs. 53/92 (57%), P = 0.40).

**Table 3:** Univariate logistic regression analysis for predictors of radiologic bronchiectasis for the entire cohort (N = 187).

	Odds Ratio	95% Confidence Intervals	P value
Age	0.98	0.94–1.01	0.202
Gender	1.21	0.54–2.83	0.64
Smoker status	1.69	0.75–4.0	0.21
Presence of ADPKD	2.49	1.1–6.1	**0.031**
Creatinine	0.98	0.73–1.24	0.88
GFR	1.0	0.98–1.01	0.92
FEV1	1.42	0.25–8.32	0.69
FVC	1.0	0.99–1.02	0.52
TLC	1.0	0.98–1.03	0.50
DLCO	0.99	0.97–1.01	0.161
Transplant Hx	1.25	0.54–2.85	0.59
Time from Transplant to Study CT	1.01	0.99–1.02	0.141

**Table 4:** Subgroup univariate logistic regression analysis for predictors of radiologic bronchiectasis in patients with ADPKD (N = 92).

	Odds Ratio	95% Confidence Intervals	P value
Age	1.02	0.98–1.07	0.27
Gender	1.86	0.68–5.33	0.23
Smoker status	3.59	1.23–12.1	**0.026**
Creatinine	0.80	0.51–1.10	0.185
GFR	1.0	0.99–1.02	0.71
FEV1	1.0	0.98–1.03	0.62
FVC	1.0	0.98–1.03	0.81
TLC	1.0	0.97–1.04	0.59
DLCO	0.98	0.95–1.01	0.28
Transplant Hx	1.89	0.68–5.34	0.22
Time from Transplant to Study CT	1.0	0.99–1.01	0.26

Distribution of summary PFT patterns and frequency of clinical pulmonary diagnoses were similar among ADPKD patients and controls (Table 5). Statistically lower percent predicted mean FEV_1_, FVC, and DLCO were noted in CKD controls (Table 1) despite increased prevalence of active smokers in ADPKD patients. Abnormal FEF 25–75 was primarily associated with concomitant obstructive physiology in those clinically diagnosed with COPD or emphysema followed by advanced restriction seen in interstitial lung disease. There were no isolated low FEF 25–75 values with normal FEV1/FVC and TLC to suggest early or small airways disease. Indications for PFT testing were no different between those with APDKD and CKD, primarily done for assessment of acute or chronic respiratory symptoms or those with known pulmonary disease (63%) followed by perioperative assessment for surgical clearance or related pulmonary complications of organ transplantation (37%). There was no difference in final clinical pulmonary diagnoses.

**Table 5:** Frequency and distribution of summary respiratory diagnoses.

	No Clinical Respiratory Disease (Group 0)	Idiopathic clinical bronchiectasis (Group 1)	All Other Airways Diseases (Group 2)	Other Respiratory (Group 3)
**ADPKD** ¶				
Radiologic Bronchiectasis (N = 19)	2	6	5	6
No bronchiectasis (N = 73)	15	0	22	36
**Control** ¶				
Radiologic Bronchiectasis (N = 9)	2	3	0	4
No bronchiectasis (N = 86)	22	0	20	44

¶ P values were not significant for distribution of clinical respiratory diagnoses between ADPKD cohort and control.

All ADPKD patients with clinical bronchiectasis (6 patients) had at least one or more years (range 14–60 months) of symptoms by the time of their CT assessment. Symptoms included productive cough, recurrent rhinosinusitis, and intermittent dyspnea. Chronic rhinosinusitis was seen in two APDKD patients with clinical bronchiectasis and none without radiologic disease, while occurring in only one patient from the CKD cohort who did not have radiologic disease. No other etiologies for bronchiectasis were evident at the time of clinical assessment. All were treated with various antibiotic and/or inhaler regimens previously or at the time of reviewed CT.

**Figure 1:**
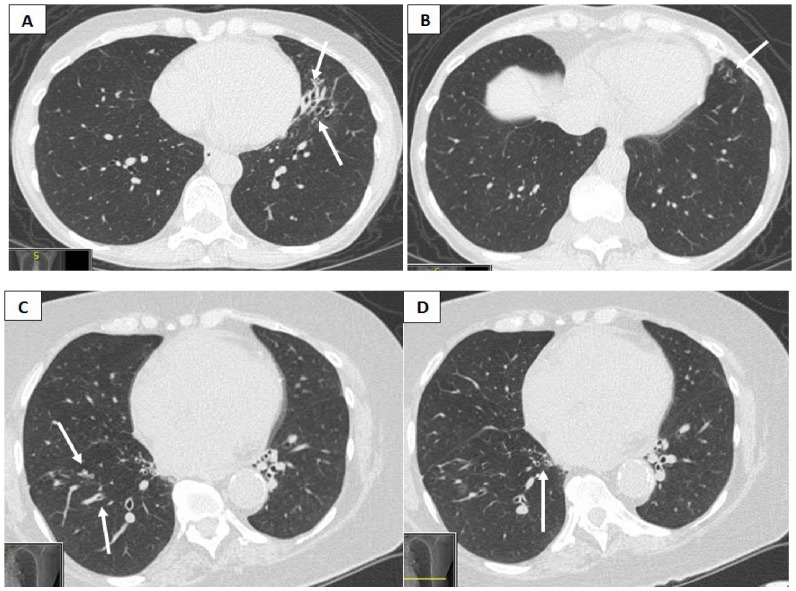
Radiologic and clinical bronchiectasis associated with ADPKD. Top panels (A and B) represent bronchiectasis in a 65 yo ADPKD female with productive cough and dyspnea on exertion, without a known secondary etiology. Panel B delineates enlarged airways visible within 1 cm of the lung periphery. Second panels (C and D) represent radiologic bronchiectasis manifesting predominantly in the right lower lobe of a 70 yo ADPKD female without clinical disease.

ADPKD-associated bronchiectasis most commonly represented mild bilateral lower lobe radiologic disease as opposed to more focal disease (Table 6) observed in control patients. Cylindrical bronchiectasis was the predominant radiological pattern in both groups (Figure 1).

**Table 6:** Location and distribution of ADPKD-associated radiologic bronchiectasis.

Lobe	Focal	Unilateral Multilobe	Diffuse (bilateral multilobe)	RUL*	RML	RLL	LUL	LML (lingula)	LLL
Group									
ADPKD (N)	7	2	10	1	6	13	0	2	13
Control (N)	6	1	2	2	3	3	1	1	4

*RUL = right upper lobe; RML = right middle lobe; RLL = right lower lobe; LUL = left upper lobe; LML = left middle lobe; LLL = left lower lobe.

## Discussion

Our study confirms previously reported [8], [16] increased prevalence of radiologic bronchiectasis associated with ADPKD (21% vs. 9%, P = 0.032). We observed similarly mild bilateral lower lobe disease using only chest CT studies. In the majority of cases, such radiologic findings were not identified during initial CT interpretation. Approximately one third of ADPKD patients with radiologic bronchiectasis (6 of 19, 32%) also had clinical idiopathic bronchiectasis however there was no difference in PFT pattern or prevalence of other pulmonary diagnoses. Finally, in ADPKD patients, smoking was associated with an increased risk of radiologic bronchiectasis (OR 4.79, CI 1.43–19.58) in our cohort even after adjustment for a priori covariates.

In the US, prevalence of clinically diagnosed bronchiectasis increases with age and ranges between 4.2 per 100,000 (age 18–34) and 271.8 cases per 100,000 individuals (age 75 and older) [17]. A recent trend analysis based on Medicare ICD-9 claims data between 2000 and 2007, found bronchiectasis prevalence to be 8.7% per year (period prevalence of 1,106 cases per 100,000 people) [18]. However, in the absence of universally accepted clinical and radiologic disease definitions, reliable prevalence studies are lacking [19], [20]. Kwak and colleagues [21] investigated the prevalence of radiologic bronchiectasis in 1409 Korean adult patients who underwent CT scanning as part of general health assessment. They reported radiologic disease in 129 (9.1%) and more than half were clinically symptomatic (53.7%) [21]. Female gender, increased age, and history of prior tuberculosis were risk factors for the presence of radiologic disease in the study. Despite comprehensive medical evaluation the cause of bronchiectasis frequently remained unidentified and such cases were subsequently classified as idiopathic. While we used a non-ADPKD CKD control cohort in our study, currently available evidence does not suggest a higher risk of radiological or clinical bronchiectasis in such patients. We did find a similar rate of radiologic bronchiectasis (9%) in our control patients as compared to the general population studied by Kwak et al.

Jain et al. recently reviewed the clinical and radiologic features of ADPKD associated bronchiectasis in 163 transplanted and non-transplanted patients [16]. They found older age as predictive of radiologic disease using a modified scoring system with the majority of presenting radiologic features similarly mild in nature. Although demographic data was not available in all studied patients, only older age was again seen as a significant risk factor for bronchiectasis based on multivariable analysis. There was also noted increased frequency and severity of radiologic disease among those with renal transplantation compared to non-transplanted patients (52.4% vs 31.4%, P = 0.02). In contrast, our study noted significant correlation of radiologic bronchiectasis with active and prior smoking history, without difference in age distribution among our more highly selected cohort using chest CT and PFT findings. Including both solid (excluding lung transplant) and hematologic organ transplants in both case and control cohorts, we noted no difference in frequency of both radiologic and clinical bronchiectasis.

The spectrum of clinical disease among those with radiologic bronchiectasis was mild to moderate at most and not statistically different between the two groups in our study. Although symptoms were reported on average greater than a year prior to selected study CT, none had history of significant weight loss, recurrent fevers, or hemoptysis. Even among those with persistent clinical symptoms, severity of radiologic disease was not advanced, with the majority of all patients with radiologic disease presenting as mild cylindrical bilateral lower lobe disease.

While the overall rate of smoking was similar between ADPKD and control patients, subgroup analysis revealed that radiologic bronchiectasis occurred more frequently in ADPKD smokers than non-smokers. This association is intriguing in the setting of known airway epithelial injury with exposure to cigarette smoke including impaired mucociliary clearance, stunned ciliary function, and decreased ciliary growth [22], [23], [24], [25], [26]. Smoking may further hasten these effects in patients with ADPKD whom perhaps have intrinsic ciliary dysfunction as compared to CKD controls whom did not see an association of radiologic bronchiectasis with smoking history in our study. Further work is needed to confirm this association while smoking cessation should be generally encouraged in all patients.

Interestingly we observed statistically lower mean FEV_1_, FVC, and DLCO among CKD patients compared to ADPKD despite comparable smoking status. As CKD patients were consecutively allocated based on presence of HRCT and PFT without matching, such findings were unlikely explained by smoking having a protective effect on PFT findings in ADPKD patients or a much more severe effect in CKD. Smoking over the entire cohort was not associated with increased radiologic or clinical bronchiectasis, but again with subgroup analysis of ADPKD patients, significantly associated with radiologic disease even after correction for a priori covariates. Summary PFT findings (normal, obstructive, restrictive, and other) were not statistically different between ADPKD and CKD patients, or among ADPKD patients with or without radiologic or clinical bronchiectasis.

A proposed mechanism for the development of bronchiectasis in patients with ADPKD may be linked to abnormalities in polycystin-1 and 2, the gene products of PKD1 and PKD 2 located on chromosomes 16 and 4 respectively. Although alterations of these genes are responsible along with other genetic abnormalities for renal cyst formation [1], [27], [28], expression of both of these genes have been reported in various cell types involved in the extra-renal manifestations of ADPKD, including valvular heart disease, vascular aneurysm, gut diverticula, and hepatic cysts [1], [28]. The presence of functional polycystin-1 in the motile cilia of human airway epithelial cells [8] and primary cilia of human airway smooth muscle cells [9] has also been demonstrated. These particular cells have been implicated in airway ciliary function and injury repair with decreased function due to polycystin abnormality, perhaps an underlying mechanism by which ADPKD patients develop radiologic and clinical bronchiectasis. The negative impact of cigarette smoke may possibly hasten these effects. Although suggestive, more directed studies are needed to confirm this mechanism at a cellular level.

Strengths of our study include the review of ADPKD patients with both high resolution chest CT (as opposed to abdominal CT with lower lung cuts) and comprehensive PFT, allowing assessment and comparison of both radiologic and functional characteristics. While our cohort provided a more specific assessment of ADPKD-associated clinical and radiologic bronchiectasis, exclusion of ADPKD and control patients whom did not undergo CT and PFT may have underestimated the true prevalence of radiologic disease, explaining our lower prevalence of radiologic findings as compared to Driscoll and colleagues [8]. As well, such selection may bias towards patients with other respiratory symptoms or pulmonary disease whose presentation may be difficult to differentiate from clinical bronchiectasis, and may not represent again a true estimate of less symptomatic bronchiectatic disease where chest CT and PFT would not have been obtained. However, our methodology was less likely to have missed ADPKD patients with more advanced or clinically relevant bronchiectasis, one of our study objectives. Another possible confounder is the inclusion of transplanted patients, whose immunosuppression may have led to increased risk of both radiologic and clinical bronchiectasis. While immunosuppression may be contributory, clinical bronchiectasis was infrequent and similar between the two groups with both groups having similar transplantation rates. As well, median time from date of transplantation to study CT was statistically similar making duration of transplantation over time less likely contributory to increased risk of infection or immunosuppression related radiologic or clinical bronchiectasis. Finally, our study has all the limitations associated with retrospective data collection and reflects the patient population of a tertiary referral center. It did allow though for maximal accrual of selected patients for a meaningful analysis using more strict radiologic and functional inclusion criteria.

In conclusion, we observed an increased prevalence of radiologic bronchiectasis among ADPKD patients who underwent high resolution chest CT. These changes most frequently involved mild-to-moderate cylindrical bronchiectasis with bilateral lower lung predominance. There were no differences in summary PFT abnormalities or frequency of clinical disease. A history of smoking in patients with APDKD may predispose to the development of radiologic and clinical bronchiectasis and smoking cessation should be generally encouraged. Radiologic bronchiectasis may be regarded as an extra-renal manifestation of ADPKD with further studies needed to explore this association.
